# Spiro­[indene-1,1′-benzo[*e*]indolin]-2′-one

**DOI:** 10.1107/S1600536810049809

**Published:** 2010-12-04

**Authors:** Jin-Xiang Chen, Yu-Qin Wang, Shu-Wen Liu, Wei-Er Lin, Zhi-Peng Chen

**Affiliations:** a School of Pharmaceutical Science, Southern Medical University, Guangzhou 510515, People’s Republic of China

## Abstract

In the title compound, C_20_H_13_NO, the indene ring is disordered over two sites with an occupancy ratio of 0.557 (2):0.443 (2). Both disordered components of indene are nearly perpendicular to the naphthalene ring system, making dihedral angles of 90.9 (2) and 85.0 (5)°. The five-membered ring of the 1*H*-pyrrol-2(3*H*)-one adopts an envelope conformation with the spiro C atom at the flap position. Inter­molecular classical N—H⋯O and weak C—H⋯O hydrogen bonding is present in the crystal structure.

## Related literature

For the biological activity of spiro lacta­ms, see: Tsuda *et al.* (2004 ▶); Chen *et al.* (2005 ▶). For the synthesis of the title compound, see: Ready *et al.* (2004 ▶); Schoemaker & Speckamp (1978 ▶).
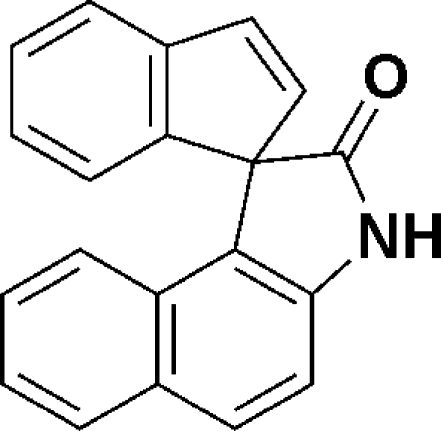


## Experimental

### 

#### Crystal data

C_20_H_13_NO
*M* *_r_* = 283.31
Monoclinic, 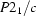

*a* = 13.0150 (18) Å
*b* = 7.9180 (11) Å
*c* = 15.537 (2) Å
β = 112.030 (2)°
*V* = 1484.2 (4) Å^3^
*Z* = 4
Mo *K*α radiation
μ = 0.08 mm^−1^
*T* = 293 K
0.50 × 0.41 × 0.33 mm

#### Data collection

Rigaku Mercury diffractometer
6913 measured reflections
2526 independent reflections
1984 reflections with *I* > 2σ(*I*)
*R*_int_ = 0.025

#### Refinement

*R*[*F* ^2^ > 2σ(*F* ^2^)] = 0.074
*wR*(*F* ^2^) = 0.199
*S* = 1.06
2526 reflections
203 parameters
30 restraints
H-atom parameters constrained
Δρ_max_ = 0.23 e Å^−3^
Δρ_min_ = −0.27 e Å^−3^

### 

Data collection: *CrystalClear* (Rigaku/MSC, 2001 ▶); cell refinement: *CrystalClear*; data reduction: *CrystalStructure* (Rigaku/MSC, 2004 ▶); program(s) used to solve structure: *SHELXS97* (Sheldrick, 2008 ▶); program(s) used to refine structure: *SHELXL97* (Sheldrick, 2008 ▶); molecular graphics: *ORTEPII* (Johnson, 1976 ▶); software used to prepare material for publication: *SHELXL97*.

## Supplementary Material

Crystal structure: contains datablocks I, global. DOI: 10.1107/S1600536810049809/xu5073sup1.cif

Structure factors: contains datablocks I. DOI: 10.1107/S1600536810049809/xu5073Isup2.hkl

Additional supplementary materials:  crystallographic information; 3D view; checkCIF report

## Figures and Tables

**Table 1:** Hydrogen-bond geometry (Å, °)

*D*—H⋯*A*	*D*—H	H⋯*A*	*D*⋯*A*	*D*—H⋯*A*
N1—H1*A*⋯O1^i^	0.86	1.99	2.815 (3)	162
C18—H18*A*⋯O1^ii^	0.93	2.35	3.064 (5)	133

Symmetry codes: (i) 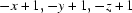; (ii) 
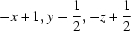
.

**Fig. 1.**
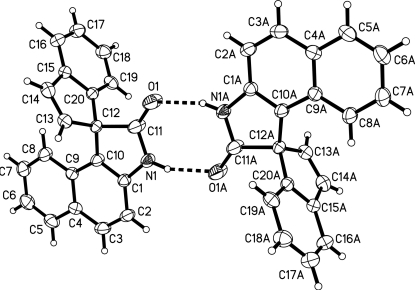
The dimer structure formed via N—H···O hydrogen bonding interactions in 1, shown as dashed lines. Symmetry transformations used to generate equivalent atoms: A: -x + 1, -y + 1, -z + 1. Hydrogen atoms are drawn as spheres of arbitrary radii.

**Fig. 2.**
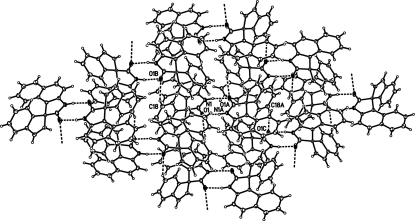
The two dimensional network structure along bc plane formed via N—H···O and C—H···O hydrogen bonding interactions in I, shown as dashed lines, Symmetry transformations used to generate equivalent atoms: A: -x + 1, -y + 1, -z + 1, B: -x + 1, y + 1/2, -z + 1/2, C: -x + 1, y - 1/2, -z + 1/2.

## supplementary crystallographic information

### Comment

In the past decades, spiro lactams have been attracted considerable interest because they are commonly found as subunits of many natural products. Some of them have significant biological activities, including multiple drug resistance reversing, antifungal and antitumor activities (Tsuda *et al.*,2004). Synthetic methods directed to these classes of spiro lactam compounds have been developed (Ready *et al.*, 2004). Among them, spiro cyclizations of *N*-acyliminium ions with an internalalkene nucleophile were first described by Speckamp (Schoemaker *et al.*, 1978). Reductive coupling of acrylates with isocyanates to furnish spiro lactam skeleton was used by Wood [Ready *et al.*, 2004]. The title compound I was synthesized in one step through a new ring-rearrangement reaction. It was undertaken as a continuation of our efforts towards synthesis of dibenzoxanthenes which exhibit a wide variety of biological activities [Chen *et al.*, 2005].

The asymmetric unit of I contains one independent spiro-[indene-1,3'-(2',3'-dihydro-2'-oxa-benzo[*e*]indole)] molecule. In the complex I, the naphthyl ring and indene ring are almost perpendicular to each other, making a diheral angle of 90.9 (2)°. All bond lengths and bond angles are in the normal ranges and comparable to those observed in the similar substituted spiro-[indene-1,3'-(2',3'-dihydro-2'-oxa-benzo[*e*]indole)], except the disordered part of indene ring. The C(11)=O(1) bond length of 1.215 (4) Å of oxa-indole moiety conforms to the value for a double bond.

In the crystal of I, there are two types hydrogen bonding interactions: one type is classical hydrogen bonding between O(1) atom and N(1) atom from oxa-benzo[*e*]indole moiety, the other one is unclassical hydrogen bonding between O(1) atom and C(18) atom from the phenyl group. The molecule I was linked together by a double strong classical intermolecular hydrogen bonds of N(1)—H1A···O(1), thereby forming a dimer structure, while the dimers were futher linked by the unclassical hydrogen bonds of C(18)—HA···O(1), thereby forming a two dimessional network structure.

### Experimental

Into a stirred solution of CuCl_2_.2H_2_O (17 mg, 0.1 mmol) in methanol (5 ml) was added a solution of ethanolamine (6 mg, 0.1 mmol) in methanol (2 ml). After 10 min. a solution of 2-amino-2'-hydroxy-1,1'-binaphthyl (0.1 mmol) in methanol (2 ml) was added and the reaction mixture was stirred at 323 K. When the reaction was completed, the solvent was removed under reduced pressure. The residue was extracted with AcOEt (10 ml), washed with 5% ammonia (10 ml) and water (10 ml), then dried with Na_2_SO_4_, and the solvent was removed under reduced pressure. Thick layer chromatography of the residue (hexane:ethyl acetate, 10:1) followed by recrystallization from acetone gave the title complex as red crystals. Yield: *ca* 82%.

### Refinement

The indene ring was found to be disordered over two sites, occupancies were refined to 0.557 (2):0.443 (2). The distance restraints have been used to make these two disordered indene ring with same bond lengths and same displacement parameters. H atoms were positioned geometrically with N—H = 0.86 and C—H = 0.93 Å, and refined using riding-model approximation with U_iso_(H) = 1.2U_eq_(C,N).

### Crystal data

**Table:**

C_20_H_13_NO	*F*(000) = 592
*M**_r_* = 283.31	*D*_x_ = 1.268 Mg m^−^^3^
Monoclinic, *P*2_1_/*c*	Mo *K*α radiation, λ = 0.71073 Å
Hall symbol: -P 2ybc	Cell parameters from 5963 reflections
*a* = 13.0150 (18) Å	θ = 2.8–25.3°
*b* = 7.9180 (11) Å	µ = 0.08 mm^−^^1^
*c* = 15.537 (2) Å	*T* = 293 K
β = 112.030 (2)°	Block, red
*V* = 1484.2 (4) Å^3^	0.50 × 0.41 × 0.33 mm
*Z* = 4	

### Data collection

**Table:**

Rigaku Mercury diffractometer	1984 reflections with *I* > 2σ(*I*)
Radiation source: fine-focus sealed tube	*R*_int_ = 0.025
graphite	θ_max_ = 24.7°, θ_min_ = 1.7°
Detector resolution: 10.0 pixels mm^-1^	*h* = −15→15
ω scans	*k* = −9→9
6913 measured reflections	*l* = −12→18
2526 independent reflections	

### Refinement

**Table:**

Refinement on *F*^2^	Primary atom site location: structure-invariant direct methods
Least-squares matrix: full	Secondary atom site location: difference Fourier map
*R*[*F*^2^ > 2σ(*F*^2^)] = 0.074	Hydrogen site location: inferred from neighbouring sites
*wR*(*F*^2^) = 0.199	H-atom parameters constrained
*S* = 1.06	*w* = 1/[σ^2^(*F*_o_^2^) + (0.0672*P*)^2^ + 1.1136*P*] where *P* = (*F*_o_^2^ + 2*F*_c_^2^)/3
2526 reflections	(Δ/σ)_max_ < 0.001
203 parameters	Δρ_max_ = 0.23 e Å^−^^3^
30 restraints	Δρ_min_ = −0.27 e Å^−^^3^

### Special details

**Table:**

Experimental. 1H NMR (CDCl_3_, 500 MHz) 8.63 (s, 1H), 7.83 (d, 1H, J = 8.5 Hz), 7.77 (d, 1H, J = 8.0 Hz), 7.56 (d, 1H, J = 7.5 Hz), 7.36 (t, 1H, J = 7.5 and 8.0 Hz), 7.32 (d, 1H, J = 5.5 Hz), 7.29 (d, 1H, J = 9.0 Hz), 7.23 (t, 1H, J =), 7.15 (t, 1H, J =), 7.11 (t, 1H, J =), 6.98 (d, 1H, J = 7.5 Hz), 6.77 (d, 1H, J = 8.5 Hz), 6.45 (d, 1H, J = 5.5 Hz); IR (KBr, cm^-1^) 3390, 3170, 3075, 2926, 1711, 1627, 1580, 1521, 1456, 1353, 1297, 1223, 814, 765, 744; Anal. C_20_H_13_ON, Calcd: C, 84.78, H, 4.62, N, 4.94, Found: C, 84.57, H, 4.65, N, 4.56.
Geometry. All e.s.d.'s (except the e.s.d. in the dihedral angle between two l.s. planes) are estimated using the full covariance matrix. The cell e.s.d.'s are taken into account individually in the estimation of e.s.d.'s in distances, angles and torsion angles; correlations between e.s.d.'s in cell parameters are only used when they are defined by crystal symmetry. An approximate (isotropic) treatment of cell e.s.d.'s is used for estimating e.s.d.'s involving l.s. planes.
Refinement. Refinement of *F*^2^ against ALL reflections. The weighted *R*-factor *wR* and goodness of fit *S* are based on *F*^2^, conventional *R*-factors *R* are based on *F*, with *F* set to zero for negative *F*^2^. The threshold expression of *F*^2^ > σ(*F*^2^) is used only for calculating *R*-factors(gt) *etc*. and is not relevant to the choice of reflections for refinement. *R*-factors based on *F*^2^ are statistically about twice as large as those based on *F*, and *R*- factors based on ALL data will be even larger.

### Fractional atomic coordinates and isotropic or equivalent isotropic displacement parameters (Å^2^)

**Table:**

	*x*	*y*	*z*	*U*_iso_*/*U*_eq_	Occ. (<1)
O1	0.4788 (3)	0.6054 (4)	0.39578 (16)	0.1336 (14)	
N1	0.3680 (2)	0.4050 (3)	0.41956 (16)	0.0717 (8)	
H1A	0.4027	0.3912	0.4783	0.086*	
C1	0.2722 (2)	0.3164 (4)	0.36566 (19)	0.0597 (7)	
C2	0.2175 (3)	0.1940 (5)	0.3960 (2)	0.0783 (10)	
H2A	0.2423	0.1625	0.4581	0.094*	
C3	0.1266 (3)	0.1220 (5)	0.3317 (3)	0.0884 (11)	
H3A	0.0890	0.0390	0.3505	0.106*	
C4	0.0870 (2)	0.1683 (4)	0.2374 (2)	0.0668 (8)	
C5	−0.0063 (3)	0.0902 (5)	0.1699 (3)	0.0896 (11)	
H5A	−0.0441	0.0067	0.1883	0.108*	
C6	−0.0415 (3)	0.1341 (5)	0.0798 (3)	0.0924 (12)	
H6A	−0.1030	0.0809	0.0367	0.111*	
C7	0.0134 (3)	0.2581 (5)	0.0508 (2)	0.0858 (11)	
H7A	−0.0117	0.2878	−0.0116	0.103*	
C8	0.1034 (3)	0.3363 (5)	0.1127 (2)	0.0793 (10)	
H8A	0.1395	0.4193	0.0923	0.095*	
C9	0.1431 (2)	0.2935 (4)	0.20776 (19)	0.0588 (7)	
C10	0.2377 (3)	0.3660 (4)	0.2759 (2)	0.0650 (8)	
C11	0.3982 (3)	0.5138 (6)	0.3676 (2)	0.1029 (15)	
C12	0.2951 (3)	0.5332 (5)	0.2738 (3)	0.0514 (10)	0.557 (2)
C13	0.2283 (5)	0.6974 (6)	0.2556 (4)	0.0639 (12)	0.557 (2)
H13	0.1859	0.7316	0.2892	0.077*	0.557 (2)
C14	0.2377 (5)	0.7833 (9)	0.1879 (5)	0.0750 (17)	0.557 (2)
H14	0.2040	0.8868	0.1666	0.090*	0.557 (2)
C15	0.3094 (3)	0.6930 (4)	0.1504 (3)	0.0611 (11)	0.557 (2)
C16	0.3462 (4)	0.7337 (5)	0.0797 (3)	0.0827 (14)	0.557 (2)
H16A	0.3242	0.8345	0.0473	0.099*	0.557 (2)
C17	0.4157 (4)	0.6237 (6)	0.0575 (3)	0.0898 (15)	0.557 (2)
H17A	0.4403	0.6509	0.0103	0.108*	0.557 (2)
C18	0.4485 (4)	0.4729 (5)	0.1060 (3)	0.0853 (15)	0.557 (2)
H18A	0.4951	0.3993	0.0911	0.102*	0.557 (2)
C19	0.4118 (4)	0.4322 (4)	0.1766 (3)	0.068 (2)	0.557 (2)
H19A	0.4337	0.3314	0.2090	0.082*	0.557 (2)
C20	0.3422 (3)	0.5422 (5)	0.1988 (2)	0.0552 (13)	0.557 (2)
C12'	0.3404 (4)	0.4514 (6)	0.2642 (3)	0.0514 (10)	0.443 (2)
C13'	0.4061 (6)	0.3612 (10)	0.2125 (5)	0.0639 (12)	0.443 (2)
H13'	0.4312	0.2503	0.2234	0.077*	0.443 (2)
C14'	0.4215 (9)	0.4607 (11)	0.1519 (9)	0.0750 (17)	0.443 (2)
H14'	0.4629	0.4323	0.1166	0.090*	0.443 (2)
C15'	0.3656 (4)	0.6226 (5)	0.1461 (3)	0.0611 (11)	0.443 (2)
C16'	0.3591 (5)	0.7675 (7)	0.0938 (4)	0.0827 (14)	0.443 (2)
H16B	0.3914	0.7695	0.0496	0.099*	0.443 (2)
C17'	0.3042 (5)	0.9094 (6)	0.1075 (4)	0.0898 (15)	0.443 (2)
H17B	0.2999	1.0064	0.0726	0.108*	0.443 (2)
C18'	0.2559 (5)	0.9064 (5)	0.1735 (4)	0.0853 (15)	0.443 (2)
H18B	0.2192	1.0014	0.1827	0.102*	0.443 (2)
C19'	0.2624 (5)	0.7615 (6)	0.2258 (4)	0.068 (2)	0.443 (2)
H19B	0.2300	0.7595	0.2699	0.082*	0.443 (2)
C20'	0.3172 (4)	0.6196 (5)	0.2121 (3)	0.0552 (13)	0.443 (2)

### Atomic displacement parameters (Å^2^)

**Table:**

	*U*^11^	*U*^22^	*U*^33^	*U*^12^	*U*^13^	*U*^23^
O1	0.148 (3)	0.160 (3)	0.0576 (14)	−0.098 (2)	−0.0021 (15)	0.0137 (16)
N1	0.0810 (17)	0.0797 (18)	0.0457 (13)	−0.0173 (14)	0.0138 (12)	0.0037 (12)
C1	0.0646 (17)	0.0605 (17)	0.0530 (16)	−0.0034 (14)	0.0209 (13)	0.0001 (13)
C2	0.086 (2)	0.089 (2)	0.0617 (18)	−0.014 (2)	0.0297 (17)	0.0139 (17)
C3	0.087 (2)	0.098 (3)	0.084 (2)	−0.028 (2)	0.035 (2)	0.011 (2)
C4	0.0625 (18)	0.0687 (19)	0.0719 (19)	−0.0075 (15)	0.0283 (15)	−0.0021 (16)
C5	0.070 (2)	0.099 (3)	0.095 (3)	−0.027 (2)	0.025 (2)	−0.006 (2)
C6	0.069 (2)	0.103 (3)	0.090 (3)	−0.020 (2)	0.0126 (19)	−0.017 (2)
C7	0.082 (2)	0.096 (3)	0.063 (2)	−0.011 (2)	0.0097 (17)	−0.0075 (19)
C8	0.083 (2)	0.084 (2)	0.0570 (18)	−0.0210 (19)	0.0111 (16)	0.0028 (17)
C9	0.0600 (16)	0.0561 (16)	0.0582 (16)	−0.0027 (14)	0.0196 (13)	−0.0016 (13)
C10	0.0726 (19)	0.0625 (18)	0.0535 (16)	−0.0158 (15)	0.0164 (14)	0.0031 (14)
C11	0.120 (3)	0.116 (3)	0.0517 (18)	−0.067 (3)	0.0086 (19)	0.0065 (19)
C12	0.057 (3)	0.046 (2)	0.0506 (19)	0.0010 (17)	0.0190 (18)	0.0007 (19)
C13	0.061 (3)	0.055 (3)	0.078 (3)	0.000 (2)	0.028 (2)	−0.006 (2)
C14	0.064 (3)	0.071 (4)	0.084 (4)	0.003 (3)	0.022 (3)	−0.006 (3)
C15	0.055 (3)	0.068 (3)	0.058 (2)	−0.004 (2)	0.019 (2)	0.007 (2)
C16	0.086 (3)	0.084 (3)	0.081 (3)	−0.005 (2)	0.034 (2)	0.024 (3)
C17	0.096 (4)	0.095 (4)	0.086 (3)	−0.007 (3)	0.042 (3)	0.012 (3)
C18	0.086 (4)	0.077 (3)	0.102 (4)	0.002 (3)	0.046 (3)	0.001 (3)
C19	0.058 (3)	0.065 (4)	0.085 (5)	−0.001 (3)	0.031 (3)	−0.013 (3)
C20	0.056 (3)	0.052 (3)	0.050 (2)	−0.010 (3)	0.0118 (18)	−0.002 (2)
C12'	0.057 (3)	0.046 (2)	0.0506 (19)	0.0010 (17)	0.0190 (18)	0.0007 (19)
C13'	0.061 (3)	0.055 (3)	0.078 (3)	0.000 (2)	0.028 (2)	−0.006 (2)
C14'	0.064 (3)	0.071 (4)	0.084 (4)	0.003 (3)	0.022 (3)	−0.006 (3)
C15'	0.055 (3)	0.068 (3)	0.058 (2)	−0.004 (2)	0.019 (2)	0.007 (2)
C16'	0.086 (3)	0.084 (3)	0.081 (3)	−0.005 (2)	0.034 (2)	0.024 (3)
C17'	0.096 (4)	0.095 (4)	0.086 (3)	−0.007 (3)	0.042 (3)	0.012 (3)
C18'	0.086 (4)	0.077 (3)	0.102 (4)	0.002 (3)	0.046 (3)	0.001 (3)
C19'	0.058 (3)	0.065 (4)	0.085 (5)	−0.001 (3)	0.031 (3)	−0.013 (3)
C20'	0.056 (3)	0.052 (3)	0.050 (2)	−0.010 (3)	0.0118 (18)	−0.002 (2)

### Geometric parameters (Å, °)

**Table:**

O1—C11	1.215 (4)	C14—C15	1.459 (6)
N1—C11	1.336 (4)	C14—H14	0.9300
N1—C1	1.401 (4)	C15—C16	1.3900
N1—H1A	0.8600	C15—C20	1.3900
C1—C10	1.353 (4)	C16—C17	1.3900
C1—C2	1.385 (4)	C16—H16A	0.9300
C2—C3	1.355 (5)	C17—C18	1.3900
C2—H2A	0.9300	C17—H17A	0.9300
C3—C4	1.407 (5)	C18—C19	1.3900
C3—H3A	0.9300	C18—H18A	0.9300
C4—C9	1.407 (4)	C19—C20	1.3900
C4—C5	1.414 (4)	C19—H19A	0.9300
C5—C6	1.346 (5)	C12'—C20'	1.528 (5)
C5—H5A	0.9300	C12'—C13'	1.549 (6)
C6—C7	1.384 (5)	C13'—C14'	1.299 (11)
C6—H6A	0.9300	C13'—H13'	0.9300
C7—C8	1.355 (4)	C14'—C15'	1.460 (8)
C7—H7A	0.9300	C14'—H14'	0.9300
C8—C9	1.411 (4)	C15'—C16'	1.3900
C8—H8A	0.9300	C15'—C20'	1.3900
C9—C10	1.410 (4)	C16'—C17'	1.3900
C10—C12	1.527 (4)	C16'—H16B	0.9300
C10—C12'	1.569 (5)	C17'—C18'	1.3900
C11—C12	1.576 (5)	C17'—H17B	0.9300
C11—C12'	1.577 (5)	C18'—C19'	1.3900
C12—C20	1.507 (5)	C18'—H18B	0.9300
C12—C13	1.530 (5)	C19'—C20'	1.3900
C13—C14	1.296 (9)	C19'—H19B	0.9300
C13—H13	0.9300		
			
C11—N1—C1	111.0 (2)	C13—C14—C15	109.6 (6)
C11—N1—H1A	124.5	C13—C14—H14	125.2
C1—N1—H1A	124.5	C15—C14—H14	125.2
C10—C1—C2	122.6 (3)	C16—C15—C20	120.0
C10—C1—N1	110.4 (3)	C16—C15—C14	131.5 (4)
C2—C1—N1	127.0 (3)	C20—C15—C14	108.5 (4)
C3—C2—C1	117.5 (3)	C15—C16—C17	120.0
C3—C2—H2A	121.2	C15—C16—H16A	120.0
C1—C2—H2A	121.2	C17—C16—H16A	120.0
C2—C3—C4	122.4 (3)	C18—C17—C16	120.0
C2—C3—H3A	118.8	C18—C17—H17A	120.0
C4—C3—H3A	118.8	C16—C17—H17A	120.0
C9—C4—C3	119.5 (3)	C17—C18—C19	120.0
C9—C4—C5	118.2 (3)	C17—C18—H18A	120.0
C3—C4—C5	122.3 (3)	C19—C18—H18A	120.0
C6—C5—C4	121.3 (4)	C20—C19—C18	120.0
C6—C5—H5A	119.3	C20—C19—H19A	120.0
C4—C5—H5A	119.3	C18—C19—H19A	120.0
C5—C6—C7	120.4 (3)	C19—C20—C15	120.0
C5—C6—H6A	119.8	C19—C20—C12	130.8 (3)
C7—C6—H6A	119.8	C15—C20—C12	109.1 (3)
C8—C7—C6	120.5 (3)	C20'—C12'—C13'	99.5 (4)
C8—C7—H7A	119.7	C20'—C12'—C10	115.4 (4)
C6—C7—H7A	119.7	C13'—C12'—C10	121.4 (4)
C7—C8—C9	120.8 (3)	C20'—C12'—C11	101.0 (4)
C7—C8—H8A	119.6	C13'—C12'—C11	121.9 (5)
C9—C8—H8A	119.6	C10—C12'—C11	96.8 (3)
C10—C9—C4	117.1 (3)	C14'—C13'—C12'	111.1 (7)
C10—C9—C8	124.2 (3)	C14'—C13'—H13'	124.4
C4—C9—C8	118.7 (3)	C12'—C13'—H13'	124.4
C1—C10—C9	121.0 (3)	C13'—C14'—C15'	111.6 (8)
C1—C10—C12	107.3 (3)	C13'—C14'—H14'	124.2
C9—C10—C12	129.3 (3)	C15'—C14'—H14'	124.2
C1—C10—C12'	106.0 (3)	C16'—C15'—C20'	120.0
C9—C10—C12'	129.1 (3)	C16'—C15'—C14'	132.9 (6)
C12—C10—C12'	34.7 (2)	C20'—C15'—C14'	107.0 (6)
O1—C11—N1	125.3 (3)	C17'—C16'—C15'	120.0
O1—C11—C12	126.6 (3)	C17'—C16'—H16B	120.0
N1—C11—C12	106.3 (3)	C15'—C16'—H16B	120.0
O1—C11—C12'	124.8 (3)	C16'—C17'—C18'	120.0
N1—C11—C12'	106.2 (3)	C16'—C17'—H17B	120.0
C12—C11—C12'	34.1 (2)	C18'—C17'—H17B	120.0
C20—C12—C10	113.7 (3)	C19'—C18'—C17'	120.0
C20—C12—C13	100.6 (4)	C19'—C18'—H18B	120.0
C10—C12—C13	119.5 (4)	C17'—C18'—H18B	120.0
C20—C12—C11	105.5 (3)	C18'—C19'—C20'	120.0
C10—C12—C11	98.6 (3)	C18'—C19'—H19B	120.0
C13—C12—C11	118.8 (4)	C20'—C19'—H19B	120.0
C14—C13—C12	112.0 (5)	C19'—C20'—C15'	120.0
C14—C13—H13	124.0	C19'—C20'—C12'	129.3 (4)
C12—C13—H13	124.0	C15'—C20'—C12'	110.6 (4)
			
C11—N1—C1—C10	0.2 (4)	C14—C15—C16—C17	179.7 (5)
C11—N1—C1—C2	180.0 (4)	C15—C16—C17—C18	0.0
C10—C1—C2—C3	0.6 (5)	C16—C17—C18—C19	0.0
N1—C1—C2—C3	−179.1 (3)	C17—C18—C19—C20	0.0
C1—C2—C3—C4	−0.5 (6)	C18—C19—C20—C15	0.0
C2—C3—C4—C9	0.1 (6)	C18—C19—C20—C12	−176.9 (4)
C2—C3—C4—C5	178.4 (4)	C16—C15—C20—C19	0.0
C9—C4—C5—C6	−0.4 (6)	C14—C15—C20—C19	−179.8 (4)
C3—C4—C5—C6	−178.7 (4)	C16—C15—C20—C12	177.5 (4)
C4—C5—C6—C7	0.0 (6)	C14—C15—C20—C12	−2.2 (4)
C5—C6—C7—C8	0.2 (6)	C10—C12—C20—C19	−51.5 (5)
C6—C7—C8—C9	0.0 (6)	C13—C12—C20—C19	179.5 (3)
C3—C4—C9—C10	0.1 (5)	C11—C12—C20—C19	55.4 (4)
C5—C4—C9—C10	−178.2 (3)	C10—C12—C20—C15	131.3 (3)
C3—C4—C9—C8	178.9 (3)	C13—C12—C20—C15	2.3 (4)
C5—C4—C9—C8	0.6 (5)	C11—C12—C20—C15	−121.8 (3)
C7—C8—C9—C10	178.3 (3)	C1—C10—C12'—C20'	−134.1 (4)
C7—C8—C9—C4	−0.4 (5)	C9—C10—C12'—C20'	68.5 (5)
C2—C1—C10—C9	−0.4 (5)	C12—C10—C12'—C20'	−36.6 (4)
N1—C1—C10—C9	179.4 (3)	C1—C10—C12'—C13'	105.7 (5)
C2—C1—C10—C12	163.6 (3)	C9—C10—C12'—C13'	−51.8 (7)
N1—C1—C10—C12	−16.6 (4)	C12—C10—C12'—C13'	−156.9 (7)
C2—C1—C10—C12'	−160.1 (3)	C1—C10—C12'—C11	−28.4 (4)
N1—C1—C10—C12'	19.7 (4)	C9—C10—C12'—C11	174.1 (4)
C4—C9—C10—C1	0.0 (5)	C12—C10—C12'—C11	69.0 (4)
C8—C9—C10—C1	−178.7 (3)	O1—C11—C12'—C20'	−54.7 (6)
C4—C9—C10—C12	−160.1 (3)	N1—C11—C12'—C20'	146.2 (4)
C8—C9—C10—C12	21.1 (6)	C12—C11—C12'—C20'	50.8 (4)
C4—C9—C10—C12'	154.6 (4)	O1—C11—C12'—C13'	54.0 (8)
C8—C9—C10—C12'	−24.2 (6)	N1—C11—C12'—C13'	−105.1 (5)
C1—N1—C11—O1	−178.9 (5)	C12—C11—C12'—C13'	159.5 (7)
C1—N1—C11—C12	15.7 (4)	O1—C11—C12'—C10	−172.2 (5)
C1—N1—C11—C12'	−19.9 (5)	N1—C11—C12'—C10	28.6 (4)
C1—C10—C12—C20	134.8 (3)	C12—C11—C12'—C10	−66.8 (4)
C9—C10—C12—C20	−63.0 (5)	C20'—C12'—C13'—C14'	3.7 (9)
C12'—C10—C12—C20	41.5 (4)	C10—C12'—C13'—C14'	131.5 (8)
C1—C10—C12—C13	−106.5 (4)	C11—C12'—C13'—C14'	−105.7 (9)
C9—C10—C12—C13	55.7 (6)	C12'—C13'—C14'—C15'	−4.1 (12)
C12'—C10—C12—C13	160.1 (6)	C13'—C14'—C15'—C16'	178.9 (6)
C1—C10—C12—C11	23.6 (4)	C13'—C14'—C15'—C20'	2.7 (11)
C9—C10—C12—C11	−174.2 (4)	C20'—C15'—C16'—C17'	0.0
C12'—C10—C12—C11	−69.8 (4)	C14'—C15'—C16'—C17'	−175.8 (8)
O1—C11—C12—C20	53.8 (6)	C15'—C16'—C17'—C18'	0.0
N1—C11—C12—C20	−141.0 (4)	C16'—C17'—C18'—C19'	0.0
C12'—C11—C12—C20	−46.1 (4)	C17'—C18'—C19'—C20'	0.0
O1—C11—C12—C10	171.5 (5)	C18'—C19'—C20'—C15'	0.0
N1—C11—C12—C10	−23.4 (4)	C18'—C19'—C20'—C12'	176.2 (5)
C12'—C11—C12—C10	71.5 (4)	C16'—C15'—C20'—C19'	0.0
O1—C11—C12—C13	−58.0 (7)	C14'—C15'—C20'—C19'	176.8 (6)
N1—C11—C12—C13	107.2 (5)	C16'—C15'—C20'—C12'	−176.9 (4)
C12'—C11—C12—C13	−157.9 (6)	C14'—C15'—C20'—C12'	−0.1 (7)
C20—C12—C13—C14	−1.7 (6)	C13'—C12'—C20'—C19'	−178.5 (4)
C10—C12—C13—C14	−126.8 (5)	C10—C12'—C20'—C19'	49.9 (6)
C11—C12—C13—C14	112.8 (5)	C11—C12'—C20'—C19'	−53.2 (5)
C12—C13—C14—C15	0.4 (7)	C13'—C12'—C20'—C15'	−2.0 (5)
C13—C14—C15—C16	−178.6 (4)	C10—C12'—C20'—C15'	−133.6 (4)
C13—C14—C15—C20	1.2 (6)	C11—C12'—C20'—C15'	123.3 (4)
C20—C15—C16—C17	0.0		

### Hydrogen-bond geometry (Å, °)

**Table:**

*D*—H···*A*	*D*—H	H···*A*	*D*···*A*	*D*—H···*A*
N1—H1A···O1^i^	0.86	1.99	2.815 (3)	162
C18—H18A···O1^ii^	0.93	2.35	3.064 (5)	133

Symmetry codes: (i) −*x*+1, −*y*+1, −*z*+1; (ii) −*x*+1, *y*−1/2, −*z*+1/2.
